# Wobble decoding by the *Escherichia coli* selenocysteine insertion machinery

**DOI:** 10.1093/nar/gkt764

**Published:** 2013-08-27

**Authors:** Jianqiang Xu, Victor Croitoru, Dorothea Rutishauser, Qing Cheng, Elias S.J. Arnér

**Affiliations:** ^1^Division of Biochemistry, Department of Medical Biochemistry and Biophysics, Karolinska Institutet, Stockholm SE-171 77, Sweden and ^2^Division of Physiological Chemistry I, Department of Medical Biochemistry and Biophysics, Proteomics Karolinska (PK/KI), Karolinska Institutet, Stockholm SE-171 77, Sweden

## Abstract

Selenoprotein expression in *Escherichia coli* redefines specific single UGA codons from translational termination to selenocysteine (Sec) insertion. This process requires the presence of a Sec Insertion Sequence (SECIS) in the mRNA, which forms a secondary structure that binds a unique Sec-specific elongation factor that catalyzes Sec insertion at the predefined UGA instead of release factor 2-mediated termination. During overproduction of recombinant selenoproteins, this process nonetheless typically results in expression of UGA-truncated products together with the production of recombinant selenoproteins. Here, we found that premature termination can be fully avoided through a SECIS-dependent Sec-mediated suppression of UGG, thereby yielding either tryptophan or Sec insertion without detectable premature truncation. The yield of recombinant selenoprotein produced with this method approached that obtained with a classical UGA codon for Sec insertion. Sec-mediated suppression of UGG thus provides a novel method for selenoprotein production, as here demonstrated with rat thioredoxin reductase. The results also reveal that the *E. coli* selenoprotein synthesis machinery has the inherent capability to promote wobble decoding.

## INTRODUCTION

**Scheme 1.**
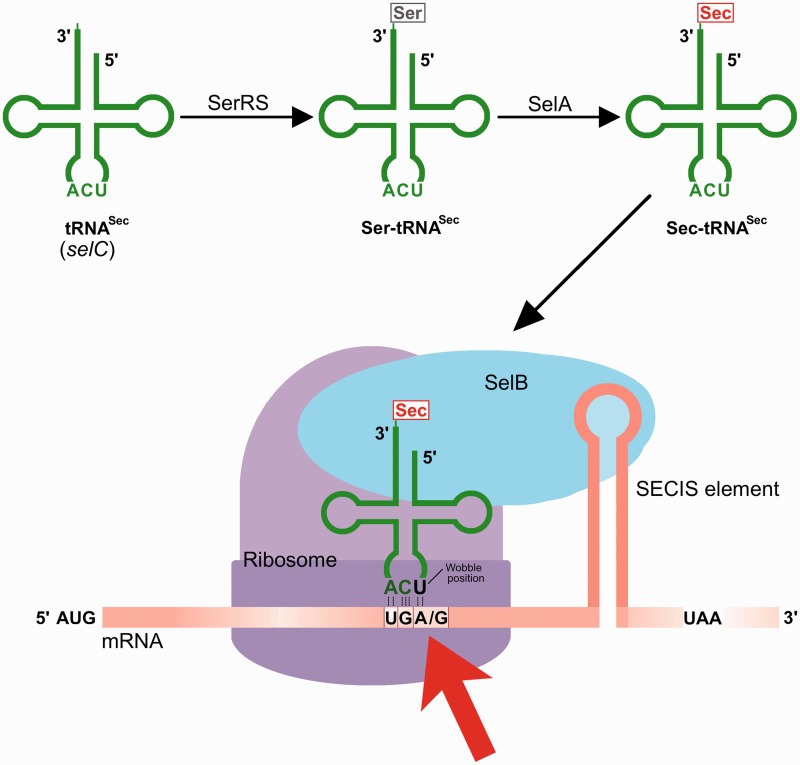
Selenoprotein expression in *E. coli* redefines a single predefined UGA codon from translational termination to Sec insertion. The Sec-encoding UGA is identified by a Sec-specific elongation factor (SelB) through interaction with an mRNA secondary structure formed by a SECIS. During expression of recombinant selenoproteins, this process typically requires Sec-tRNA^Sec^ (the *selC* gene product) that is amino acylated by seryl-tRNA-synthetase (SerRS) with the seryl moiety converted to selenocysteinyl by Sec synthase (the *selA* gene product). Here, we demonstrate high-yield SECIS-dependent wobble decoding with Sec-mediated suppression of a UGG codon (red arrow) using this synthesis machinery.

Selenocysteine (Sec) is the 21st amino acid and defining entity for selenoproteins (1). It is a sulfur-to-selenium substituted analogue of cysteine (Cys), with higher chemical reactivity and unique properties compared with Cys (2,3). The high reactivity of Sec likely explains why Sec-specific selenoprotein synthesis machineries tightly control Sec synthesis and its translational incorporation into selenoproteins. During selenoprotein expression, the genetic code is redefined in a unique process whereby specific Sec-encoding UGA codons are diverted from their natural roles of serving as translational termination signals (4). This redefining process requires a *cis*-acting secondary structure in the selenoprotein mRNA—a so-called Sec Insertion Sequence (SECIS) element (5)—that is recognized by a specialized Sec insertion machinery (6). This includes the unique Sec-specific SelB elongation factor binding the SECIS element and catalyzing Sec insertion at the UGA; several properties of Sec insertion machineries however differ between different domains of life (4,7,8). In *Escherichia coli,* the machinery requires the products of the *selA*, *selB*, *selC* and *selD* genes (1,4) (Scheme 1) that compete in their UGA decoding processes with translational termination through release factor 2 (RF2) (9) and, under certain conditions, tryptophan (Trp)-mediated UGA suppression (10,11). Recombinant selenoprotein production in *E. coli* therefore typically results in mixtures of products with either Sec insertion or RF2-mediated truncation products (12,13), and in some cases Trp insertion (11). In this study, we asked whether the Sec insertion machinery itself could support wobble decoding, which has not been investigated to date, although it is a common phenomenon with classical amino acids (14). Indeed, we found Sec-mediated wobble decoding to be possible, and, when using overproduction of the *selA*, *selB* and *selC* genes together with UGG decoding, it allowed for recombinant selenoprotein production in *E. coli*.

Two non-SECIS-dependent methods for recombinant production of selenoproteins in *E. coli* were recently reported, using either mutations in 16S rRNA (15) or synthetic tRNA species compatible with the classical elongation factor EF-Tu (16). Those methods show promise for production of selenoproteins having internal Sec residues, where the presence of a SECIS element is a complicating factor. However, when a Sec residue is located close to the C-terminal end of the protein, such as in isoenzymes of thioredoxin reductase (TrxR) (12,17,18) or Sel-tagged proteins (19–21), high-yield production is enabled by the engineering of a synthetic SECIS element that is not itself translated but still compatible with the bacterial Sec insertion machinery for support of Sec insertion close to the C-terminus (22). This technique however results in a mixture of Sec-insertion and RF2-mediated UGA truncation (23,24). Here, we analyzed whether truncation can be avoided through Sec-mediated wobble decoding and used production of rat TrxR1 because its Sec contents at position 498 is easily probed through analyses of its enzymatic activity (13).

## MATERIALS AND METHODS

### TrxR1 expression plasmids

Wild-type rat TrxR1 was expressed as previously described, using a recombinant system enabling incorporation of the active site Sec with the used plasmid pET-TRS_TER_ containing a bacterial-type SECIS (12). The truncated TrxR1 variant missing both Sec^498^ and Gly^499^ was generated as described previously (25,26) with the bacterial-type SECIS element in the plasmid (5′-ggttgcaggtctgcacc-3′; 17 nt) and furthermore replaced with a non-SECIS sequence (5′-agaatcactagtgcggc-3′; 17 nt) to yield a pET-TRS_TER (-SECIS)_ plasmid. Either pET-TRS_TER_ or pET-TRS_TER (-SECIS)_ was used as PCR templates to construct the different mutants of rat TrxR1 used in this study. The primers used are shown in Table 1, and molecular cloning operations were performed as described (27,28). DNA sequencing (GATC Biotech, Konstanz, Germany) verified the mutations. For recombinant expression, the plasmids were transformed into either *E. coli* BL21 (DE3) *gor^−^*cells (Tet^+^) with or without accessory pSUABC plasmid (12) (Chl^+^) or its derivatives, as indicated.

**Table 1.** Primers used for UGA-to-UGG substitutions

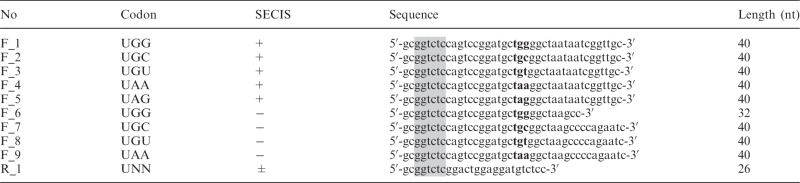

**Figure 1.**
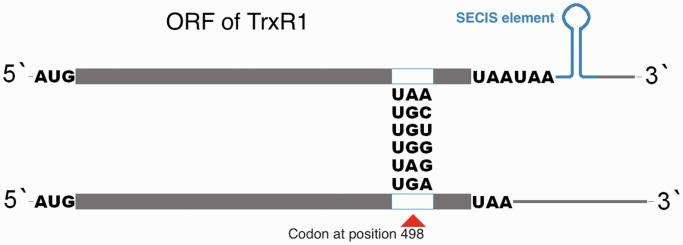
Scheme of the herein used constructs. The plasmid pET-TRS_TER_, designed with a UGA codon at the position 498 corresponding to Sec and a SECIS element at its 3′-UTR for production of recombinant TrxR as a selenoprotein 1, was used as a PCR template for site-directed mutagenesis (for primers, see Table 1). Five codons including UGG (encoding Trp), UGC or UGU (both encoding Cys), UAG (amber stop codon), or UAA (ochre stop codon), were made to replace the UGA codon. The stem-loop structure in blue indicates the functional SECIS element, recognized by SelB as required to catalyze Sec insertion. Regarding constructs made to encode truncated enzyme or other variants lacking a SECIS element (DNA sequence being 5′-ggttgcaggtctgcacc-3′), this was replaced with a non-SECIS encoding sequence (5′-agaatcactagtgcggc-3′), and an additional UAA codon was introduced at position 500 (as indicated in the lower cartoon of the scheme).

This table lists the primers used for introduction of mutations at the codon corresponding to Sec within the open reading frame of rat TrxR1, with or without a downstream SECIS element (see scheme in Figure 1). The nucleotides in bold denote the codon change, whereas the shaded nucleotides introduce restriction cleavage sites for cloning. The R_1 primer was used as a reverse primer in all PCR reactions.

N, represents G, A, U or C.

### Variant SelC and SelABC expression plasmids

Plasmid pCDF-selC-UCA (2505 bp) was constructed by subcloning an *Xba* I/*Acc*65 I fragment (397 bp, from pSUABC) containing *selC* gene (91 bp) into *Xba* I/*Acc*65 I linearized pCDF-1b (Novagen, USA). The lac I region and T7 promotor region of pCDF-1b were removed by digestion with *Xba* I and *Acc*65 I. Then, plasmid pCDF-selC-UCA was used as a PCR template for mutating *selC* gene at the anticodon of the Sec-specific tRNA^Sec^_(UCA)_ (WT) from UCA to either CCA or CUA, and finally resulted in pCDF-selC-CUA and pCDF-selC-CCA. The primers used are given in Table 2. The additional pSUABC-derived plasmids pSUABC-CCA, pSUABC-UCA and pSUABC-CUA were subsequently constructed by removing the standard *selC* cassette from the standard pSUABC using *Xba* I and *Sac* I and replacing it with the *Xba* I/*Sac* I *selC* fragment from the corresponding pCDF-selC plasmids. Two new primers (Table 2) were synthesized for DNA sequencing (GATC Biotech, Konstanz, Germany), which verified the mutations on pCDF-selC. For recombinant expression, the plasmids were transformed into either *E. coli* BL21 (DE3) *gor^−^* cells (Tet^+^) with or without accessory pSUABC plasmid (Chl^+^) or pCDF-selC plasmid (Sm^+^) as indicated in the text.

**Table 2.** Primers used for *selC* (tRNA^Sec^) mutations

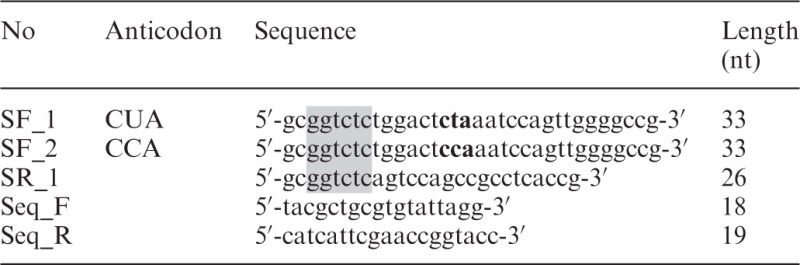

This table lists the primers used for introduction of mutations at the anticodon of tRNA^Sec^ (the *selC* gene product), using the pCDF-selC-UCA plasmid as template. The nucleotides in bold denote the anticodon change, whereas the shaded nucleotides introduce restriction cleavage sites for cloning. The SR_1 primer was used as the reverse primer in both PCR reactions, whereas the two Seq primers were used for DNA sequencing verification of the resulting products.

### Protein expression and purification

All rat TrxR1 variants were recombinantly expressed in *E. coli* BL21 (DE3) *gor^−^* host strains, with or without co-transformation with the pSUABC plasmid, according to previously described methods (12). The ‘2.4/24/24’ protocol was used to facilitate production of wild-type rat TrxR1 as previously described (23) with all the variants produced under the same conditions for comparison reasons. After 2′,5′-ADP Sepharose^TM^ (GE Healthcare Life Sciences, Uppsala, Sweden) column purification, samples were concentrated over 30 kDa cutoff Centrifugal Filters (Ultracel^TM^ YM-30, Millipore, MA, USA) and thereupon sterile-filtered over 0.20 μm autoclaved filter membranes. For each protein, 5 ml of concentrated samples were subsequently loaded onto a gel filtration column Superdex^TM^ 200 GL 10/300 (120 ml, GE Healthcare Life Sciences, Uppsala, Sweden) pre-equilibrated with 50 mM Tris–HCl, 2 mM EDTA (pH 7.5) (TE) buffer. The dimeric enzyme species were collected and pooled analyses and activity assays. The >99% pure enzymes were desalted after the gel filtration column and stored in TE buffer at 4°C. All protein purifications were performed at 4°C using an ÄKTA Explorer 100 workstation (GE Healthcare Life Sciences, Uppsala, Sweden) with absorbance tracking at 280 nm (protein absorbance) and 463 nm (flavin adenine dinucleotide [FAD] detection). Flavoprotein concentrations of purified enzymes were determined using the FAD absorbance at 463 nm (with the extinction coefficient of 11 300 M^−^^1 ^cm^−^^1^), and protein concentration was estimated using a Bradford assay Kit (Bio-Rad, USA) with bovine serum albumin as standard. For purity analyses, the TrxR1 samples were separated on NuPAGE^TM^ 4–12% Bis-Tris SDS–PAGE gels (Life Technologies, USA) on heating at 70°C in SDS Loading buffer (Life Technologies, USA) containing 20 mM dithiothreitol for 10 min. The protein bands were visualized by staining with Coomassie Brilliant Blue R250 (GE Healthcare Life Sciences, Uppsala, Sweden) and documented using a Bio-Rad ChemiDoc XRS scanner (Bio-Rad, USA).

### ^75^Se autoradiography

Bacteria were grown at 37°C for 12 h until an OD_600nm_ = 2.4 whereupon temperature was lowered to 24°C. For radiolabeling, 100 μg ml^−^^1^ of l-Cys (to avoid non-specific ^75^Se incorporation into sulfur metabolism), 5 μM cold sodium selenite and 1 μCi [^75^Se]-selenite (Missouri Research Reactor, USA) were added into the culture medium. Thereupon, 0.5 mM Isopropyl β-D-1-thiogalactopyranoside (IPTG) was added to induce protein expression, which was allowed for another 24 h of growth at 24°C with constant aeration in rotating culture flasks. Thereafter, cells were collected and lyzed with 1 mg ml^−^^1^ lysozyme and ultrasonication whereupon samples clarified supernatants on centrifugation at 16 000 g for 30 min were used for analyses with reducing SDS–PAGE gel and autoradiography with documentation using a GE Typhoon^TM^ FLA 7000 Biomolecular Imagers and ImageQuant^TM^ TL software version 7.0 (GE Healthcare Life Sciences, Uppsala, Sweden).

### TrxR activity assays

Enzymatic activities of the TrxR1 variants were determined using three separate assays: the standard 5,5′-dithiobis-(2-nitrobenzoic acid) (DTNB) reduction assay (29), an insulin-coupled Trx reduction assay (29), or a phenanthrenequinone reduction assay (30). The experimental details in 96-well microtiter plate format are as follows: (i) DTNB reduction assay. The standard reaction mixture (200 μl) contained 2.5 mM DTNB, 17 nM TrxR variant enzyme and 300 μM NADPH in 50 mM TE buffer, pH 7.5. DTNB reduction was evaluated by the formation of TNB^−^ following the absorbance at 412 nm for 10 min, with the extinction coefficient of 13 600 M^−^^1 ^cm^−^^1^. (ii) Insulin-coupled Trx reduction assay. The standard reaction mixture (200 μl) contained 20 μM hTrx1, 160 μM insulin, 170 nM TrxR variant enzyme and 300 μM NADPH in 50 mM TE buffer (pH7.5). Activity assay was performed following the NADPH consumption as decrease of A340 nm using an extinction coefficient of 6200 M^−^^1 ^cm^−^^1^. (iii) Phenanthrenequinone reduction assay. The standard reaction mixture (200 μl) in this assay contained 50 μM phenanthrenequinone, 17 nM enzyme and 200 μM NADPH in 50 mM TE buffer (pH 7.5), and activity was measured by following NADPH consumption at 340 nm. All the activity assays were performed with 10 s time interval reads at 25°C using a VersaMax microplate reader (Molecular Devices, USA), with the reaction mixtures without enzyme serving as reference. Activity measurements were performed in at least duplicate and analyzed with the Prism 5 software (GraphPad, USA).

### Electrospray Ionization mass spectrometry

For analyses with mass spectrometry, buffer exchange of the enzyme samples to 10 mM ammonium bicarbonate (NH_4_HCO_3_) buffer (pH7.7) was performed using NAP-25^TM^ column (GE Healthcare Life Sciences, Uppsala, Sweden) and further concentrated to final concentrations of 40 μM using 30 kDa cut-off Centrifugal Filter devices (Ultracel^TM^ 30K, Millipore, MA, USA). Aliquots of 2 μl of the cleaned proteins were diluted in 20 μl of 50% ACN/H_2_O and 20 μl of 0.5% HCOOH. Data were acquired on a QTOF Premier API mass spectrometer (Waters, Milford, US) equipped with a Z-spray source, operated in the positive ion mode under the control of MassLynx 4.1. Samples were introduced via a nanoflow electrospray interface from metal-coated borosilicate glass capillary needles (EconoTips™, New Objective), and scans were acquired between *m*/*z* 500 and 2000 with a rate of 1 scan/s and an interscan interval of 0.1 s. The source temperature was set to 80°C, capillary voltage to 3.3 kV, cone and RF lens energies to 40 and 50 V, respectively. Cone gas 85 was set to and Nano gas to 0.22. The instrument was operated in single-reflector mode at a resolution of 10 000 (full width at half-maximum definition). The mass scale was calibrated against [Glu1]-fibrinopeptide B. Mass spectra were analyzed using Waters MassLynx software and MaxEnt1 for deconvolution of the data.

## RESULTS

### IPTG-induced production of Sec-containing TrxR1 using alternative Sec-encoding codons

**Figure 2.**
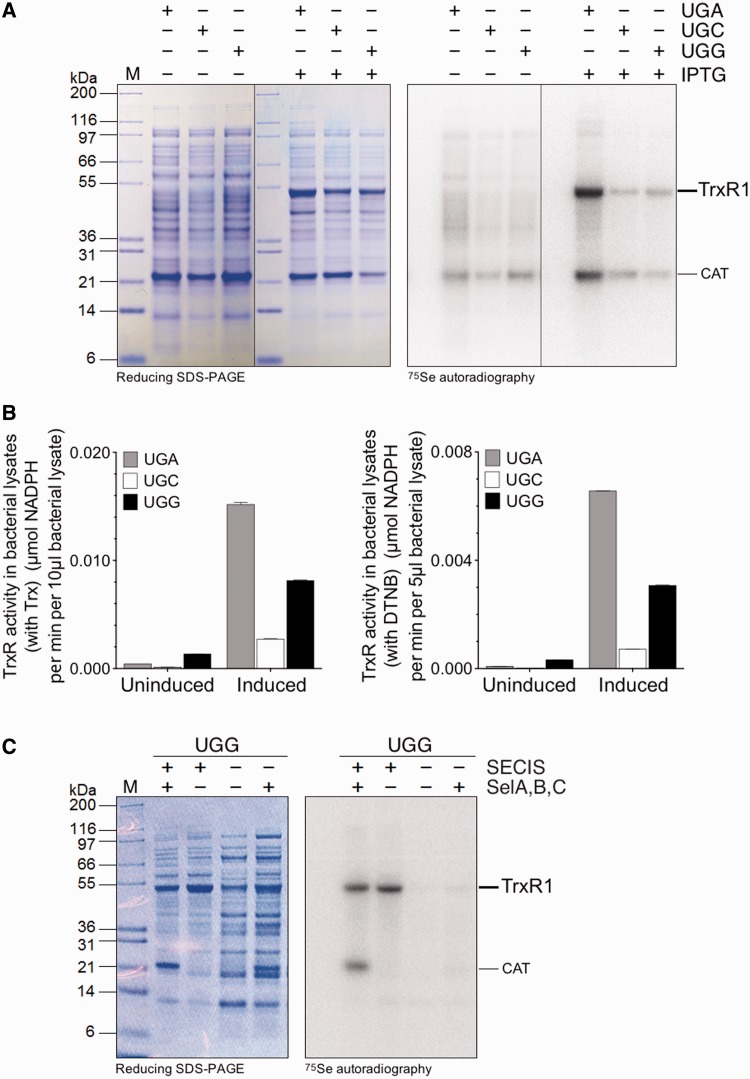
Sec-mediated suppression of UGG in *E. coli* using the bacterial Sec insertion machinery. Detection of selenoprotein production by ^75^Se radiolabeling and SDS–PAGE analysis as well as TrxR1 activity assays on cell lysates were used to screen for selenoprotein production, using conditions indicated in the figure: with (‘Induced’) or without (‘Uninduced’) IPTG induction of recombinant TrxR1production, with either UGA, UGC or UGG codon at position 498 corresponding to Sec in the native selenoprotein. All expression conditions were made using a bacterial-type SECIS element in the construct and overproduction of the *selA*, *selB* and *selC* genes. (**A**) A Coomassie-stained SDS–PAGE analysis with the corresponding autoradiography is shown in the left panel, with the position of the TrxR1 subunit size indicated. (**B**) TrxR1-specific activities in crude cell lysates using either a Trx-linked assay or direct DTNB reduction. (**C**) Production of TrxR1 using constructs with a UGG codon at the position corresponding to Sec in the enzyme was performed with or without a SECIS element or overexpression of the *sel* genes and visualized using ^75^Se radiolabeling, as indicated. The diffuse radioactive band seen just above ≈21 kDa on contransformation with pSUABC, together with expression of ^75^Se-labeled TrxR1, may be due to unspecific labeling of the pSUABC plasmid resistance gene, chloramphenicol acetyl transferase (indicated in the figure as ‘CAT’).

Because a full TrxR1 activity is dependent on the presence of a Sec residue in its C-terminal active site (12,13), we first assessed whether active recombinant TrxR1 could be expressed in *E. coli* utilizing constructs having either UGA, UGC or UGG codons at the position corresponding to Sec (see Figure 1 for a scheme of the different constructs used in this study). We found a notable IPTG-inducible ^75^Se-labeled protein expression and TrxR1 activity not only using a UGA codon (Sec/Stop) but also with UGG (Trp) or, to a lesser extent, UGC (Cys) (Figure 2A and B). The ^75^Se incorporation with UGG still required the presence of a SECIS element in the construct, thus implying selenoprotein synthesis machinery-dependent Sec-mediated wobble decoding at the single UGG codon located directly upstream of the SECIS element (Figure 2C).

### Enzymatically active recombinant TrxR1 can be produced using several different Sec-defining codons, with UGG being the best alternative to UGA

**Figure 3.**
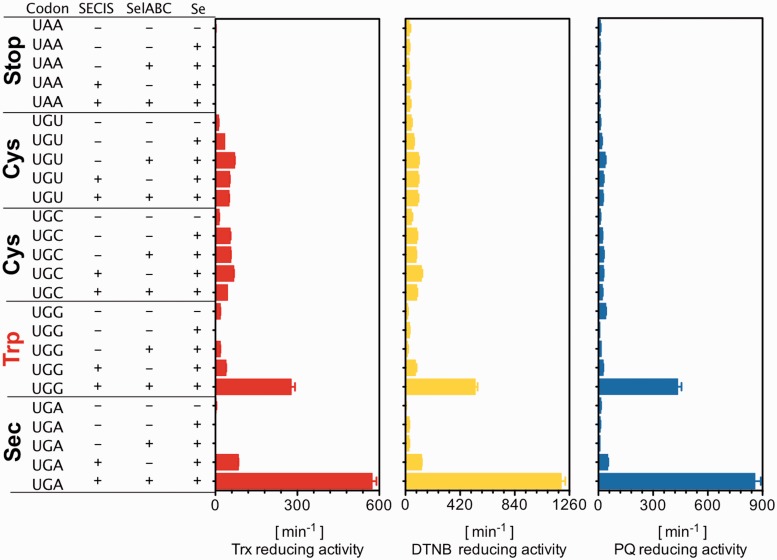
Identification of Sec-mediated UGG-encoded TrxR1 products in *E. coli*. Expression of TrxR1 from constructs having either UAA (Stop), UGU (Cys), UGC (Cys), UGG (Trp) or UGA (Stop/Sec) at the position of Sec in the wild-type enzyme were performed in *E. coli* either with or without a SECIS element in the construct, overexpression of the *sel* genes and supplementation of the medium with selenite, as indicated in the figure. The 25 enzyme variants were purified to apparent homogeneity (Figure 6) whereupon turnover in three separate TrxR1-specific assays was determined (Trx1-coupled insulin reduction, direct DTNB reduction or PQ reduction), as indicated in the figure and further described in the text.

To further analyze the properties of these recombinant TrxR1 variants, we expressed and purified to apparent homogeneity the proteins made from constructs using either of four codons at the position corresponding to Sec (UAA, UGG, UGU, UGA or UGG), with or without the presence of a SECIS element. The protein variants were furthermore expressed under conditions of either selenium supplementation in the medium (5 μM sodium selenite) and/or overexpression of the bacterial *selA*, *selB* and *selC* genes. The latter was previously shown to increase yield in expression of recombinant TrxR1 as a selenoprotein when UGA is used as the Sec-encoding codon (17,18). Using three distinct well-defined assays for measurement of TrxR1 activity, i.e. Trx-coupled insulin reduction (22), direct DTNB reduction (22) or direct phenanthrene quinone (PQ) reduction (30), we found that the TrxR1 protein expressed with a UGG codon at the position of Sec, in the presence of a SECIS element and together with overexpression of the *sel* genes, produced an enzyme having approximately half the specific activity as compared with that using a native UGA codon, when expressed and purified under identical conditions (Figure 3).

### Complementary anticodon of tRNA^Sec^ does not necessarily improve yield

**Figure 4.**
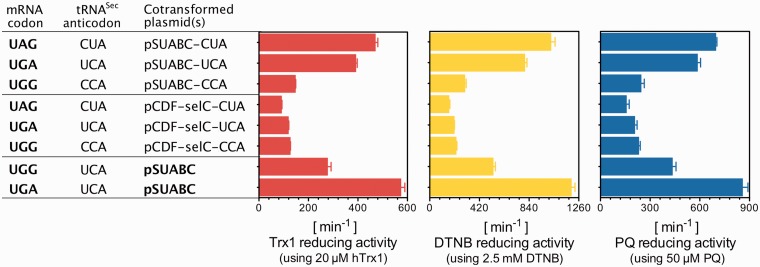
Effect of the Sec-specific tRNA^Sec^ variants and codon combinations on TrxR1 production in *E. coli*. By changing the natural anticodon UCA of tRNA^Sec^ to either CCA or CUA, we analyzed its support of Sec insertion using expression of TrxR1 from constructs having either UAG (Stop), UGA (Stop/Sec) or UGG (Trp) at the position of Sec. Expression was performed in conjunction with a SECIS element in the construct, overexpression of the *sel* genes (pSUABC) or only the *selC* gene (pCDF-selC), and supplementation of the medium with selenite, as indicated in the figure. As for details of expression yields using the cotransformed plasmid(s), please see Table 3. The eight enzyme variants were purified to apparent homogeneity (Figures 6 and 7) whereupon turnover in three TrxR1-specific assays was determined (Trx1-coupled insulin reduction, direct DTNB reduction or PQ reduction), as indicated in the figure and further described in the text.

We next asked whether the specific activity of TrxR1, and thus its Sec contents, when produced using a UGG codon could be further improved by mutation of the anticodon of the *selC*-encoded tRNA^Sec^ from UCA to CCA, to obtain an exact match to the UGG. Interestingly, this change of the tRNA^Sec^ lowered the specific activity of the produced enzyme, probably because of less efficient usage of this non-natural tRNA in one or several steps of the selenoprotein synthesis cascade (16,29). However, it was possible to instead use UAG amber codon suppression in place of UGA, thereby allowing selenoprotein synthesis to compete with RF1 instead of RF2, especially when used in a context of a perfect codon–anticodon pair. That strategy resulted in production of TrxR1 having about the same specific activity as when using native UGA-directed Sec insertion (Figure 4).

### Validation of recombinant TrxR1 variants using mass spectrometry for identification of the penultimate amino acid

**Figure 5.**
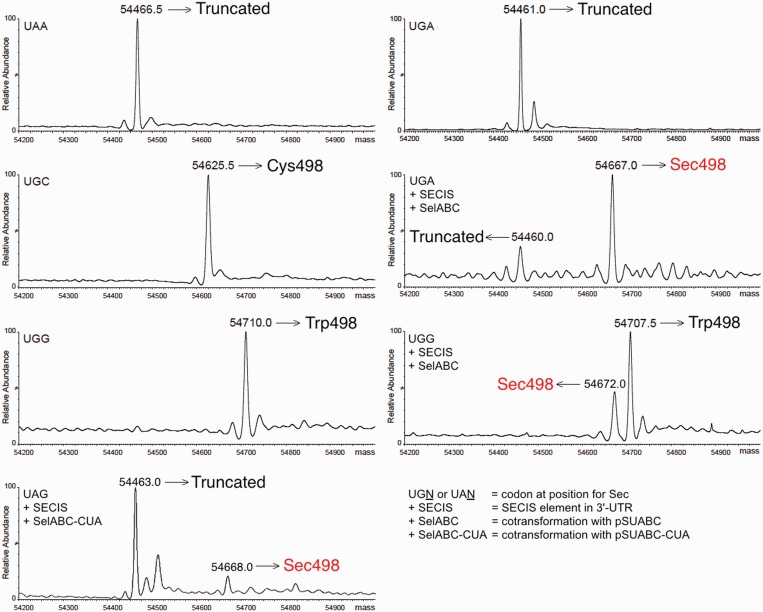
ESI-Q-TOF mass spectrometry of variant TrxR1 forms produced in *E. coli*. The purified TrxR1 variants expressed from constructs having either UAA, UGC, UGG or UGA at the position corresponding to Sec were analyzed with Q-TOF-MS, as described in the ‘Materials and Methods’ section. Deconvoluted zero-charged ions at average mass are shown (expected accuracy ±0.01% ≈±5.5 Da for TrxR1). When the UGG codon variant was expressed in conjunction with a SECIS element, *sel* gene overexpression and selenite supplementation, the product contained a mixture of the Sec-to-Trp substituted enzyme (Trp498, 54 707 Da) and wild-type Sec-containing protein (54 672.0 Da, indicated in red), without any trace of the truncation product seen for enzyme produced with UGA or UAA at the position of Sec (54 461 Da).

To further verify the identities of the TrxR1 species indirectly probed for Sec contents through enzyme activity measurements, we performed ESI-Q-TOF mass spectrometry analyses of all protein species within the range of 54–55 Da (zero-charged average mass) as found in the enzyme preparations (Figure 5). These analyses confirmed that the nature of the purified enzyme species were as we had presumed. This included enzyme produced with UAA at the position corresponding to Sec (solely truncated protein, theoretical mass 54 464.39 Da), with UGC in the absence of a SECIS element (solely Cys-containing protein, theoretical mass 54 624.50 Da), with UGG in a construct without a SECIS element (Trp-containing variant, theoretical mass 54 707.70 Da) or with UGA but without a SECIS element (again solely truncated enzyme). It was also clear, as expected, that the enzyme expressed from a construct containing a SECIS element together with *sel*gene overexpression and selenite supplementation, having a UGA at the position of Sec, gave a major peak of 54 667 Da corresponding to the theoretical size of wild-type TrxR1 (54 671.48 kDa) mixed with a peak of 54 460 Da corresponding to UGA-truncated enzyme. The analyses, finally, confirmed that the UGG codon variant expressed in the presence of a SECIS element with *sel* gene overexpression and selenite supplementation, resulted in a product containing a mixture of Sec-to-Trp substituted enzyme and wild-type Sec-containing protein (54 672 Da, indicated in red in Figure 5), without any trace of a truncation product. The enzyme preparation produced using UAG in place of UGA at the Sec-encoding position together with a tRNA^Sec^ mutated to a complementary anticodon also clearly contained the full-length Sec-containing TrxR1 enzyme, in addition to truncated enzyme (Figure 5, lower left panel). These results showed that the identity of the TrxR1 variants agreed well with the expected results. As a final analysis, we next wished to evaluate all of the production strategies for TrxR1 as studied here, with regards to final yields and specific activities when produced and purified under identical conditions.

### High specific activities and total yields of recombinant selenoprotein TrxR1 can be achieved using either UGA, UAG or UGG as the Sec-defining codon

**Figure 6.**
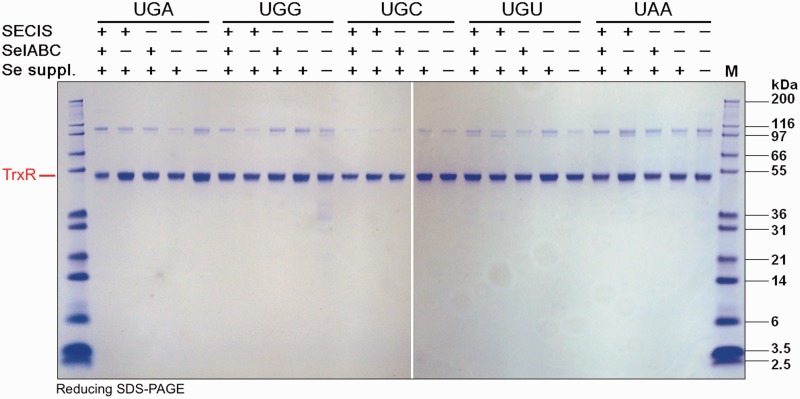
Purity analysis of purified TrxR variants. The TrxR1 variants produced using the indicated codons at position 498 were recombinantly expressed in BL21 (DE3) *gor*^−^ host strains and further spurified over 2′,5′-ADP Sepharose^TM^ affinity chromatography followed by Superdex^TM^ G-200 gel filtration chromatography (GE Healthcare Life Sciences, Uppsala, Sweden). The purified protein samples were here analyzed on reducing SDS–PAGE gels. ‘M’ stands for M12 protein standards (Life Technologies, USA), with size (kDa) indicated in the figure. The strong ≈55 kDa protein bands represent the resolved TrxR1 subunits, whereas the weak ≈110 kDa bands are traces of covalently linked TrxR1 dimers stable in reducing SDS–PAGE, typically seen in analyses of this enzyme.

**Figure 7.**
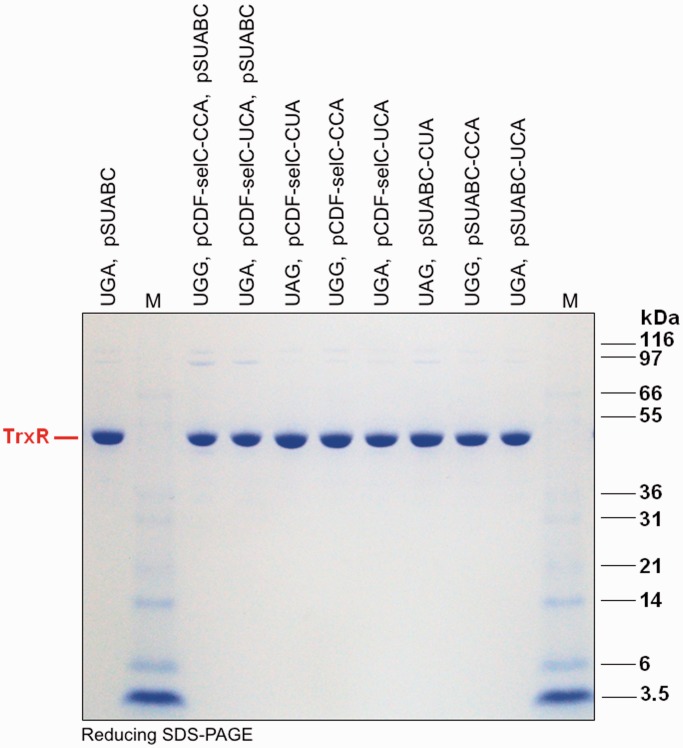
Purity analysis of additional TrxR variants produced using alternative tRNA^Sec^ variants. Using co-transformations with the pCDF-selC plasmid and/or pSUABC together with the pET-TRS_TER_ TrxR1 expression plasmid in *E. coli* BL21 (DE3) *gor*^−^ host strains, these TrxR1 were recombinantly expressed and further purified over 2′,5′-ADP Sepharose^TM^ affinity chromatography followed by Superdex^TM^ G-200 gel filtration chromatography (GE Healthcare Life Sciences, Uppsala, Sweden). Sodium selenite was supplemented at 5 μM in the bacterial medium for all productions. The purified protein samples were here analyzed on reducing SDS–PAGE gels. ‘M’ stands for M12 protein standards (Life Technologies, USA), with size (kDa) indicated in the figure. The strong ≈ 55 kDa protein bands represents the resolved TrxR1 subunits, whereas the weak ≈110 kDa bands are traces of covalent TrxR1 dimers stable in reducing SDS–PAGE, as typically seen in analyses of this enzyme.

For the final analyses of efficiency in Sec insertion and versatility for production of TrxR1 as a selenoprotein, we produced all of the variants of TrxR1 studied herein using 1 l of bacterial cultures, with identical production and purification procedures, with determination of specific activities and final yields. All the variants could be purified with rather high yields with regards to protein amount and at high apparent purity, when using either alternative codons for Sec with or without the SECIS and the pSUABC plasmid (Figure 6), or in combinations with novel variants of tRNA^Sec^ (Figure 7). Determining the specific activities and total yields of these TrxR variants, we found that, under the conditions used in the present study, production of TrxR1 using either native UGA at position 498 together with the native tRNA^Sec^, or the UAG amber codon together with a complementary anticodon in the tRNA^Sec^, yielded specific activities of TrxR1 at 10–15 U/mg, with total yields of 308 to 448 U from 1 l of bacterial culture. Using UGG together with the native selenoprotein synthesis machinery resulted in a specific activity of 6.3–6.8 U/mg and yields of 191–195 U/l culture. None of the other production strategies used herein, with regards to choice of Sec-defining codon, presence of SECIS element or tRNA^Sec^ variant usage, gave any specific activities or total yields that could approach these values (Table 3).

**Table 3.** Specific activities and total yields of TrxR1 variants purified from 1 l cultures

Enzyme variants and production parameters						TrxR variants Yield and specific activity^a^		
Variants at position 498 of TrxR1	mRNA codon^b^	tRNA^Sec^ anticodon^c^	SECIS^d^	Selenite^e^	Contransformed plasmid(s)^f^	mg	U mg^−1,^^g^	U l^−1^ culture
Stop	UAA	UCA	−	−	−	17.2	0.36	6.28
	UAA	UCA	−	+	−	27.8	0.38	10.5
	UAA	UCA	−	+	pSUABC	20.6	0.33	6.82
	UAA	UCA	+	+	−	23.7	0.42	9.94
	UAA	UCA	+	+	pSUABC	24.2	0.42	10.2

Cys	UGU	UCA	−	−	−	27.0	0.55	14.8
	UGU	UCA	−	+	−	28.3	0.81	22.9
	UGU	UCA	−	+	pSUABC	19.4	1.3	24.5
	UGU	UCA	+	+	−	16.7	1.2	20.5
	UGU	UCA	+	+	pSUABC	15.6	1.2	18.5

Cys	UGC	UCA	−	−	−	34.8	0.58	20.2
	UGC	UCA	−	+	−	33.5	1.1	37.1
	UGC	UCA	−	+	pSUABC	34.5	1.0	35.5
	UGC	UCA	+	+	−	20.5	1.6	31.8
	UGC	UCA	+	+	pSUABC	26.9	1.1	29.3

Trp	UGG	UCA	−	−	−	7.84	0.22	1.72
	UGG	UCA	−	+	−	17.6	0.39	6.85
	UGG	UCA	−	+	pSUABC	16.1	0.20	3.21
	UGG	UCA	+	+	−	18.3	0.98	17.9
	UGG	UCA	+	+	pSUABC	28.3	6.8	191

Sec/Stop	UGA	UCA	−	−	−	19.0	0.03	0.57
	UGA	UCA	−	+	−	27.9	0.36	9.98
	UGA	UCA	−	+	pSUABC	23.8	0.35	8.35
	UGA	UCA	+	+	−	16.8	1.6	26.1
	UGA	UCA	+	+	pSUABC	22.5	15	339

Stop	UAG	CUA	+	+	pSUABC-CUA	34.6	13	448
Sec/Stop	UGA	UCA	+	+	pSUABC-UCA	30.4	10	308
Trp	UGG	CCA	+	+	pSUABC-CCA	30.9	3.8	116
Stop	UAG	CUA	+	+	pCDF-selC-CUA	30.3	2.1	62.1
Sec/Stop	UGA	UCA	+	+	pCDF-selC-UCA	17.3	2.6	45.5
Trp	UGG	CCA	+	+	pCDF-selC-CCA	33.7	2.8	95.1
Sec/Stop	UGA	UCA+UCA	+	+	pSUABC, pCDF-selC-UCA	27.0	7.6	205
Trp	UGG	UCA+CCA	+	+	pSUABC, pCDF-selC-CCA	30.9	6.3	195

All TrxR1 protein variants studied here were expressed under identical conditions in 1 l of cultures and subsequently purified to apparent near-homogeneity (see Figures 6 and 7 for Coomassie-stained SDS–PAGE analyses of the final purifications). This table summarizes the final yields and specific activities for these TrxR1 variants.

^a^Final yields and activities of purified dimeric TrxR variants as purified from 1 l of bacterial culture.

^b^In-frame codon corresponding to position 498 of TrxR1 (natural variant encoding Sec/Stop is UGA).

^c^The identity of the anticodon of the Sec-specific tRNA^Sec^ *selC* gene product (natural variant is UCA).

^d^Presence (+) or absence (−) of a bacterial-type SECIS element in the construct.

^e^Presence (+) or absence (−) of sodium selenite (5 µM) supplementation to the bacterial growth medium.

^f^Plasmids cotransformed with the TrxR1-encoding pET plasmid.

^g^Specific activity as determined in the standard DTNB reduction assay (29).

## DISCUSSION

In this study, we found that a single predefined UGG codon can be redirected for Sec incorporation into a recombinantly expressed selenoprotein, using efficient wobble decoding mediated by the *E. coli* selenoprotein synthesis machinery. We furthermore found that premature termination at the Sec-defining codon is thereby avoided; instead, yielding a mixture of Trp and Sec insertion in contrast to the UGA-mediated truncation typically seen in overproduction of selenoproteins. These findings reveal a hitherto unknown flexibility in the *E. coli* selenoprotein synthesis machinery that may be of importance for both a full understanding of the bacterial Sec insertion mechanisms, as well as for future use in biotechnological applications based on production of recombinant selenoproteins.

We indirectly examined the efficiency of Sec insertion through the production of enzymatically active TrxR1, using several different Sec-defining codons in place of the native UGA, with or without the SECIS element or overexpression of the *selA*, *selB* and *selC* genes. We also analyzed the effects of anticodon mutations in tRNA^Sec^ (the *selC* gene product). Several of the results are noteworthy, as they yield further insights into the fidelity or specificity of the bacterial Sec insertion machinery. First, it is clear that very little, if any, selenoprotein was produced in absence of a bacterial-type SECIS element, irrespective of codon choice. This result could have been expected, as it is in full agreement with the early work of Böck and coworkers first deciphering the selenoprotein synthesis machinery of *E. coli* (1,4,5). The finding underscores that any Sec insertion that is not mediated through SelB interacting with the SECIS element, is virtually non-existent, under the production conditions used herein. The finding that by changing the in-frame UGA codon to UAG, concurrent with mutation of the anticodon of tRNA^Sec^ to a complementary CUA, yielded about the same expression of selenoprotein TrxR1 as using the native UGA/UCA combination, was also in agreement with early findings by Böck *et al.*, having shown similar results in other reporter systems (31). The findings were also reminiscent to results of Berry *et al.*, who showed that mutations of UGA to either UAA or UUA abolished production and enzymatic activities of recombinantly expressed Sec-dependent thyroid hormone deiodinases in mammalian cells, whereas complementary mutations of a contransfected tRNA^Sec^ could, at least partially, restore their production (32). Indeed, match pairing between a codon and the anticodon of the corresponding tRNA is, naturally, important for the decoding process during translation. With tRNA^Sec^-mediated Sec insertion, more factors are however of crucial importance and may help explain some of the other results in our attempts to express Sec-containing TrxR in the present study. Importantly, the product of the *selC* gene, i.e. the Sec-specific tRNA^Sec^, is originally aminoacylated with serine, which is later converted to Sec by replacement of the side chain hydroxyl group with a selenol, in an intricate process catalyzed by SelA (33–36). In the recent crystal structure of a SelA/tRNA^Sec^ complex, there is no evidence for interactions between SelA and the anticodon of tRNA^Sec^ (34)*.* Thus, the selenocysteinylation of tRNA^Sec^ is likely to be independent of the nature of the anticodon. It is thereby plausible that some other step in the selenoprotein synthesis pathway should explain why our mutation of the anticodon of tRNA^Sec^ to CCA, when used as a perfect match to UGG, ‘lowered’ Sec incorporation as compared with the wobble decoding of the UGG codon using the native tRNA^Sec^. However, this UGG wobble decoding in itself was a novel finding, as we are not aware of any prior studies having described Sec-mediated wobble decoding of a defined UGG (Trp) codon, using SECIS-guided insertion. However, we only achieved good yields when both the selenoprotein-encoding mRNA and the *selA*, *selB* and *selC* genes were overexpressed. Overexpression of these key mediators of selenoprotein synthesis was previously shown to increase the Sec insertion (12,37), which probably relates to the fact that intracellular stoichiometry in the mRNA/SelB/tRNA^Sec^ complex needs to be maintained (38). Our results reveal that these criteria are valid also when the selenoprotein translation machinery mediates wobble decoding of UGG.

The highly intricate machinery for selenoprotein synthesis makes Sec insertion the limiting factor for production of recombinant selenoproteins at high yields, even if growth or culture conditions that lower the competing RF2-mediated translational termination can somewhat improve final yields, such as expression at late exponential phase (23). Still, the basic methodology used by us for synthesis of selenoproteins in *E. coli*, with an engineered SECIS element variant compatible with the bacterial machinery (12), has in some ways become a cornerstone production method for production of selenoproteins having their Sec residue located close to the C-terminal end of the protein (18,20,39). One direct approach for lowering RF2-levels clearly increases Sec incorporation specificity, but at the cost of a lower growth rate and reduced total yield, which has hampered widespread use of that strategy (40). Other researchers have developed alternative methods for selenoprotein synthesis, including synthetic production (41), use of novel tRNA species (16) or orthogonal ribosomes (15). Our production of TrxR1 using a UAG codon for Sec coupled with the pSUABC-CUA plasmid encoding tRNA^Sec^(CUA) resembled the utilization of UAG for incorporation of Sec in GPx coupled with the new tRNA^Ser^(CUA) variant recognized by EF-Tu (16), with the difference that in our study the original *selC*-encoded tRNA^Sec^(UCA) scaffold was used and thus required SelB-mediated insertion in concert with a SECIS element. All the currently available methods for selenoprotein production still have limitations and will typically result in either low yields or in products being mixtures of the target selenoprotein with non-Sec-containing protein species. The methodologies of our present study, using Sec-mediated wobble decoding at alternative codons to the natural UGA, with especially good yields at UGG (resulting in Sec/Trp mixtures in the final product) or at UAG (competing with translational termination by RF1 instead of RF2), provide additional production strategies. These may prove useful as alternative approaches for recombinant selenoprotein production and, furthermore, illustrate the inherent capability of the *E. coli* selenoprotein synthesis machinery to suppress UGG with Sec, provided that a UGG codon is positioned within a functional context of a bacterial-type SECIS element.

## FUNDING

ESJA from the Swedish Cancer Society, the Swedish Research Council (Medicine) and Karolinska Institutet. Funding for open access charge: Swedish Research Council and Karolinska Institutet.

*Conflict of interest statement.* None declared.
